# Comparison of neoadjuvant chemotherapy or chemoradiotherapy plus immunotherapy for locally resectable esophageal squamous cell carcinoma

**DOI:** 10.3389/fimmu.2024.1336798

**Published:** 2024-05-08

**Authors:** Guozhen Yang, Haodong Yue, Xiaomin Zhang, Chufeng Zeng, Linyu Tan, Xu Zhang

**Affiliations:** ^1^ Department of Thoracic Oncology, Sun Yat-sen University Cancer Center, Guangzhou, China; ^2^ Guangdong Esophageal Cancer Institute, Guangzhou, China; ^3^ State Key Laboratory of Oncology in South China, Guangdong Provincial Clinical Research Center for Cancer, Sun Yat-sen University Cancer Center, Guangzhou, China; ^4^ Department of Pathology, Sun Yat-sen University Cancer Center, Guangzhou, China; ^5^ School of Nursing, Sun Yat-sen University, Guangzhou, China

**Keywords:** esophageal squamous cell carcinoma, neoadjuvant therapy, immunotherapy, chemotherapy, chemoradiotherapy

## Abstract

**Background:**

Neoadjuvant chemotherapy plus immunotherapy (nCT + ICIs) and neoadjuvant chemoradiotherapy plus immunotherapy (nCRT + ICIs) both induced favorable pathological response and tolerant toxicities for locally resectable esophageal squamous cell carcinoma (ESCC). However, few studies compared safety and efficacy between the two treatment strategies.

**Methods:**

This retrospective study collected clinical data of locally resectable ESCC patients who underwent nCT + ICIs or nCRT + ICIs followed by esophagectomy from November 2019 to December 2022. The incidence of adverse events, surgical outcomes, short and long-term efficacy, and treatment costs were compared.

**Results:**

A total of 206 patients were included, with a ratio of 158:48 between nCT + ICIs group and nCRT + ICIs group. The two groups exhibited well-balanced baseline characteristics. Most adverse events were grade 1-2 in both groups. The nCT + ICIs group had a longer operative time (334.00 ± 170.2 min vs 279.60 ± 88.31 min, *P*=0.020) than nCRT + ICIs group, but there were no differences in surgical complications. Although nCT + ICIs group had a lower pCR rate (32.3% vs 52.1%, *P*=0.004), the 2-year overall survival (84.42% vs 81.70%, *P*=0.860), 2-year disease-free survival (83.21% vs 80.47%, *P*=0.839), and recurrence patterns were similar to nCRT + ICIs group. In addition, nCT + ICIs group had significantly lower expenses (188796.00 ± 107704.00 RMB vs 231808.00 ± 48067.00 RMB, *P*=0.045).

**Conclusion:**

Overall, nCT + ICIs have comparable safety and efficacy compared to nCRT + ICIs for locally resectable ESCC, but with lower hospitalization costs.

## Introduction

Esophageal cancer is the 7^th^ most common malignancy and the 6^th^ leading cause of cancer-related death over the word (1). In China, more than 32,0000 new confirmed cases and 30,0000 new deaths in 2022, ranks the 6^th^ in incidence and 4^th^ in mortality (2). More than 90% are esophageal squamous cell carcinoma (ESCC). Esophagectomy is the primary treatment for locally resectable ESCC. However, the 5-year overall survival (OS) is only 15%-30% with surgery alone. Based on the NEOCRTEC5010 clinical trial (3), neoadjuvant chemoradiotherapy followed by esophagectomy has become the primary treatment for locally resectable ESCC, and improve the 5-year OS to 59.9%. However, the latest long-term follow-up results (4) indicated that 34.6% of patients still experience treatment failure due to postoperative recurrence, especially the rate of distant metastasis as high as 25.3%. Thus, the current neoadjuvant treatment strategies are still far from the clinical needs. It needs to explore more effective comprehensive treatment modalities.

Immunotherapy, especially the advent of immune checkpoint inhibitors (ICIs), has advanced the treatment prospects for many advanced tumors, including ESCC. Now, programmed cell death 1 (PD-1) inhibitor plus chemotherapy has been approved for the first-line treatment of advanced ESCC based on the appreciable outcomes in ESCORT-1^st^ (5) and Checkmate648 (6). For locally resectable ESCC, when compared to postoperative adjuvant therapy, preoperative neoadjuvant therapy can effectively eliminate tumor cells before surgery, reduce the risk of recurrence and metastasis, and be better tolerated by patients. Thus, in recent years, increasing studies have focused on neoadjuvant immunotherapy for locally resectable ESCC. The existing neoadjuvant immunotherapy modalities primarily consist of two approaches: neoadjuvant chemoradiotherapy plus immunotherapy (nCRT+ICIs) and neoadjuvant chemotherapy plus immunotherapy (nCT+ICIs). An exploratory study reported by Lee et al. at the 2019 ASCO was the first to investigate the effectiveness and safety of nCRT+ICIs. In this study, 28 cases of ESCC were included, receiving pembrolizumab combined with platinum-based chemoradiotherapy. Although the pathological complete response (pCR) rate reached 46.1%, two patients experienced acute lung injury (7). Subsequently, in the PALACE-1 study, the pCR rate after nCRT+ICIs even reached 55.6% (8). However, pneumonia remains one of the common and clinically challenging adverse events after radiotherapy (9). Therefore, clinical trials of nCT+ICIs have been initiated. In a pilot study conducted by Zhigang Li et al. (10), which enrolled 60 cases, investigated the feasibility of carrelizumab plus chemotherapy followed by surgery for locally resectable ESCC. The results indicated pCR rate could reach to 39.2%, and adverse events were well-tolerated. Both of these neoadjuvant models demonstrated high pCR rates and excellent clinical prospects. However, the current trials were small-sample, single-arm studies. There is no research directly comparing the safety and effectiveness of different preoperative immunotherapy models to determine the best neoadjuvant immunotherapy treatment program.

Therefore, this study aimed to compare the differences in safety, short and long-term efficacy and hospitalization costs between nCT + ICIs and nCRT + ICIs.

## Materials and methods

### Patient selection

ESCC patients who underwent neoadjuvant immunotherapy combined with chemotherapy or chemoradiotherapy followed by esophagectomy from November 2019 to December 2022 were retrieved from Sun Yat-sen University Cancer Center database. Inclusion criteria consisted of 1) age 18-80 years old, and 2) thoracic squamous cell carcinoma confirmed on pathology, and 3) received at least one cycle of PD-1 blockade combined with chemotherapy or chemoradiotherapy, and 4) clinical stage T1N1-3 or T2-4aN0-3 according to the American Joint Committee on Cancer (AJCC), 8th edition, and 5) Eastern Cooperative Oncology Group (ECOG) performance score ≤ 1, and 6) patients with complete demographics, clinical information, and postoperative follow-up data. The main exclusion criteria included non-squamous cell carcinoma, patients who received neoadjuvant targeted therapy, patients with advanced or metastatic tumor assessed as unresectable, and patients with incomplete clinical information. This study was permitted by the Ethics Committee of Sun Yat-sen University Cancer Center.

### Drugs regimens during neoadjuvant treatment

Patients in the nCT + ICIs group were given 1-6 cycles of intravenous PD-1 inhibitor and simultaneous chemotherapy. The PD-1 inhibitor contained camrelizumab, pembrolizumab, sintilimab, tislelizumab, toripalimab and penpulimab. The chemotherapy regimens contained paclitaxel-based drugs plus platinum-based drugs, nab-paclitaxel plus capecitabine or S1, and nab-paclitaxel alone. The median treatment duration was 3 cycles. The detailed treatment regimens are presented in **Supplementary Figure 1**. The dosage and usage of the medicine can be seen in **Supplementary Table 1**.

Patients in the nCRT + ICIs group were treated with 2-3 cycles of intravenous PD-1 inhibitor combined with concurrent chemoradiotherapy. The PD-1 inhibitor included tislelizumab, toripalimab, pembrolizumab, sintilimab and nivolumab. The chemotherapy regimens contained paclitaxel-based drugs plus platinum-based drugs. All patients received radiotherapy as the institutional protocol. The median immunotherapy duration was 2 cycles. The detailed treatment regimens are also presented in **Supplementary Figure 1**. The dosage and usage of the medicine can be seen in **Supplementary Table 1**.

### Surgery procedure

Esophagectomy is performed after 4-8 weeks from the completion of the last neoadjuvant treatment. Patients received McKeown or Ivor-Lewis minimally invasive esophagectomy (MIE), with or without robotic-assistance. Traditional thoracotomy was also considered for patient with severe adhesion. Routine two-field lymph node dissection was carried out, and three-field lymph node dissection was performed for patients with preoperative suspicion of cervical lymph node metastasis. All surgeries were conducted by experienced thoracic surgeons.

### Follow-up

Patients were followed up every 3 months during the initial year, and subsequently every 6-12 months in the following years. Physical examination, contrast-enhanced computed tomography (CT), barium scans and cervical and supraclavicular lymph node ultrasonography were regularly performed when patients returned to hospital for review. Patients with dysphagia or anastomotic fistula were considered for esophagoscopy, and those suspected of distant metastasis underwent positron emission tomography/computed tomography (PET-CT). Patient follow-up data were obtained by reviewing outpatient records or through phone correspondence.

### Study endpoint

The primary endpoint was the pCR rate. The secondary endpoints included treatment-related adverse events, surgical complications, tumor regression grade (TRG), objective response rate (ORR), OS, disease-free survival (DFS) and economic efficiency analysis. And pCR was defined as the complete disappearance of viable tumor cells under microscope. TRG was evaluated based on National Comprehensive Cancer Network criteria. Grade 0 indicated the absence of viable residual tumor cells, grade 1 was residual small clusters cancer cells, grade 2 was residual cancer foci with stromal fibrosis, and grade 3 was minimal or no tumor cell regression. Tumor response assessment accorded to the Response Evaluation Criteria in Solid Tumors (11) (RECIST version 1.1). Economic efficiency analysis comprised hospitalization duration, ICU stay duration, chest drainage duration, 30-day readmission rate, 90-day readmission rate, and overall treatment expenditure. OS was defined as the duration between surgery and death from any cause or loss to follow-up. DFS was defined as the duration between surgery and disease recurrence.

### Statistical analysis

All data were subjected to analysis using R Version 4.0.4 (R Foundation for Statistical Computing, Vienna, Austria). Categorical variables were presented as frequencies (%), while continuous variables were presented as median ± standard deviation. The categorical variables were analyzed using the chi-squared test (χ² test) or Fisher’s exact test. Inter-group comparisons of continuous variables following a normal distribution were conducted using the t-test, while for those not following a normal distribution, the Mann-Whitney U test was employed. In order to control the potential biases resulting from differences in clinical factors between the nCT + ICIs and nCRT + ICIs groups, a multivariable logistic regression model or multiple linear regression model was used to adjust for age, gender, tumor location, differentiation, ECOG performance status, clinical T stage, clinical N stage, smoking history, alcohol consumption history, chemotherapy regimen, and cycles of neoadjuvant treatment. Survival analysis was conducted using the Kaplan-Meier method, and differences in survival curves were compared by Log-rank test. We employed Cox proportional hazards model for both univariate and multivariate analysis of clinical factors potentially associated with prognosis. *P*-value less than 0.05 on both sides were considered statistically significant.

## Results

### Baseline characteristics

**Figure 1:**
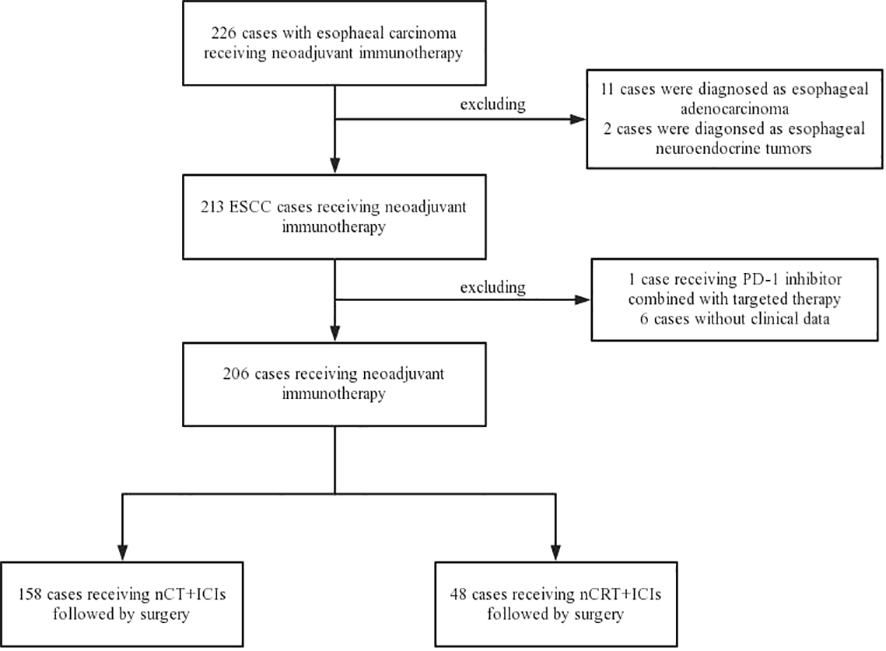
The flowchart of enrolled patients.

From November 2019 to December 2022, a total of 226 locally resectable esophageal carcinoma patients who underwent neoadjuvant immunotherapy followed by esophagectomy were identified at Sun Yat-sen University Cancer Center. Patients with non-squamous cell carcinoma and those without available follow-up information were excluded, eventually enrolled 206 locally resectable ESCC patients for analysis. Among them, 158 were assigned to the nCT + ICIs group, and 48 were assigned to the nCRT + ICIs group. The flowchart of patient enrollment was depicted in **Figure 1**. Both groups exhibited excellent baseline characteristics matching, including sex (*P*=0.632), age (*P*=0.214), tumor location (*P*=0.819), smoking history (*P*=0.468), alcohol consumption history (*P*=0.250), body mass index (BMI) (*P*=0.677), ECOG performance status (*P*=0.140), differentiation (*P*=0.174), tumor length (*P*=0.323), clinical stage T (*P*=0.068), clinical stage N (*P*=0.357), clinical stage TNM (*P*=0.063), surgical approach (*P*=0.905), extent of lymph node dissection (*P*=0.597), anastomotic position (*P*=0.954), and route of gastric conduit (*P*=0.597). The baseline clinical characteristics were presented in **Table 1**.

**Table 1:** Baseline characteristics of the patients.

Characteristics	nCT + ICIs (n=158)	nCRT + ICIs (n=48)	*P* value
Sex			0.632
Male	133 (84.2%)	39 (81.2%)	
Female	25 (15.8%)	9 (18.8%)	
Age (years old)			0.214
≦60	76 (48.1%)	28 (58.3%)	
>60	82 (51.9%)	20 (41.7%)	
Tumor location			0.819
Upper thoracic	9 (5.7%)	2 (4.2%)	
Middle thoracic	98 (62%)	32 (66.7%)	
Lower thoracic	51 (32.3%)	14 (29.2%)	
Smoking history			0.468
Yes	103 (65.2%)	34 (70.8%)	
No	55 (34.8%)	14 (29.2%)	
Alcohol consumption history			0.250
Yes	61 (38.6%)	23 (47.9%)	
No	97 (61.4%)	25 (52.1%)	
BMI (kg/m2)			0.677
≦18.5	24 (15.2%)	6 (12.5%)	
>18.5 and ≦23.9	101 (63.9%)	34 (70.8%)	
>23.9	33 (20.9%)	8 (16.7%)	
ECOG			0.140
0	101 (63.9%)	25 (52.1%)	
1	57 (36.1%)	23 (47.9%)	
Differentiation			0.174
Well differentiated	9 (5.7%)	6 (12.5%)	
Moderately differentiated	111 (70.3%)	28 (58.3%)	
Poorly differentiated	38 (24.1%)	14 (29.2%)	
Tumor length			0.323
≦50mm	76 (48.1%)	27 (56.2%)	
>50mm	82 (51.9%)	21 (43.8%)	
Clinical stage T			0.068
T1	1 (0.6%)	3 (6.2%)	
T2	41 (25.9%)	9 (18.8%)	
T3	106 (67.1%)	34 (70.8%)	
T4	10 (6.3%)	2 (4.2%)	
Clinical stage N			0.357
N0	18 (11.4%)	2 (4.2%)	
N1	64 (40.5%)	19 (39.6%)	
N2	57 (36.1%)	18 (37.5%)	
N3	19 (12%)	9 (18.8%)	
Clinical stage TNM			0.063
I	1 (0.6%)	2 (4.2%)	
II	37 (23.4%)	5 (10.4%)	
III	95 (60.1%)	30 (62.5%)	
IVA	25 (15.8%)	11 (22.9%)	
Surgical approach			0.905
MIE	152 (96.2%)	47 (97.9%)	
Thoracotomy	6 (3.8%)	1 (2.1%)	
Lymph node dissection			0.597
Two-field	142 (89.9%)	45 (93.8%)	
Three-field	16 (10.1%)	3 (6.2%)	
Anastomotic position			0.954
Cervical	157 (99.4%)	47 (97.9%)	
Thoracic	1 (0.6%)	1 (2.1%)	
Route of gastric conduit			0.597
Posterior mediastinal	142 (89.9%)	45 (93.8%)	
Restro-sternal	16 (10.1%)	3 (6.2%)	

### Treatment-related adverse events

**Figure 2:**
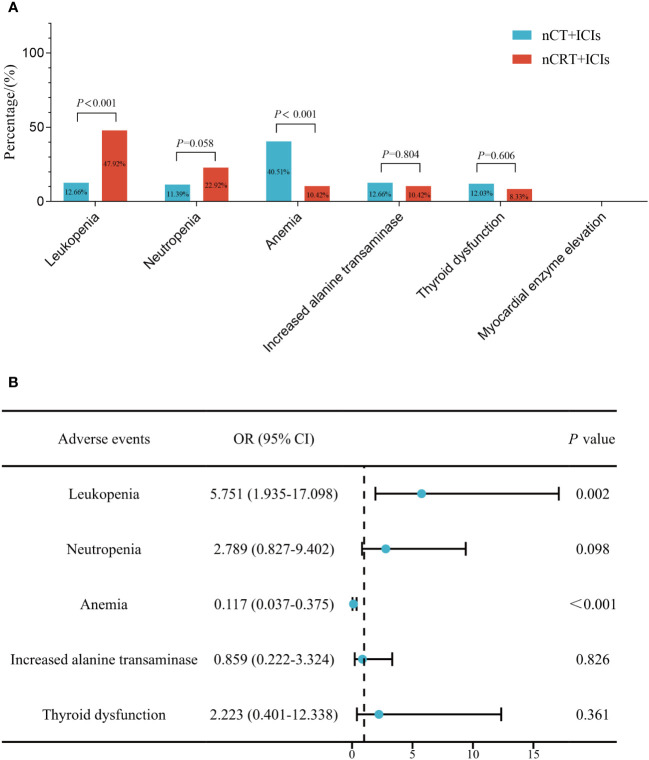
The comparison of treatment-related adverse events between nCT + ICIs group and nCRT + ICIs group. **(A)** Before adjustment for clinical factors. **(B)** After adjustment for clinical factors.

**Figure 2A** provided a summary of adverse events during neoadjuvant treatment in the two groups. In nCT + ICIs group, the most common adverse events were anemia (40.51%, 64/158), followed by increased transaminases (12.66%, 20/158). In nCRT + ICIs group, the most common adverse events were leukopenia (47.92%, 23/48) and neutropenia (22.92%, 11/48). When comparing the two groups, anemia was more common in nCT + ICIs group (*P*<0.001), while leukopenia was more common in nCRT + ICIs group (*P*<0.001). Other adverse events, such as increased transaminases and thyroid dysfunction, showed no intergroup differences. Subsequently, a multivariable logistic regression model was used to adjust for age, gender, tumor location, differentiation, ECOG performance status, clinical T stage, clinical N stage, smoking history, alcohol consumption history, chemotherapy regimen, and cycles of neoadjuvant treatment (**Figure 2B**). The results showed that the incidence of anemia remained significantly lower in the nCRT + ICIs group compared to the nCT + ICIs group (OR: 0.117, 95%CI: 0.037-0.376, *P*<0.001), while the incidence of leukopenia was still higher (OR: 5.751, 95%CI: 1.935-17.098, *P*=0.002). Regarding the severity of adverse events, grade 1-2 leukopenia and neutropenia were more frequent in nCRT +ICIs group (*P*=0.002, *P*=0.043, respectively), whereas grade 1-2 anemia was more frequent in nCT + ICIs group (*P*<0.001). Grade 3 or higher adverse events occurred at rates of 6.33% (10/158) and 8.33% (4/48) in the two groups, respectively, with no statistically significant difference (*P*=0.451). The summary of adverse events during the neoadjuvant period were provided in **Table 2**.

**Table 2:** Summary of treatment-related adverse events.

Adverse events	nCT + ICIs (n=158)	nCRT + ICIs (n=48)	*P* value	Adjusted *P* value*
Leukopenia				
Grade 1-2	16 (10.13%)	21 (43.75%)	<0.001	0.002
Grade 3-4	4 (2.53%)	2 (4.17%)	0.625	0.718
Neutropenia				
Grade 1-2	10 (6.33%)	10 (20.83%)	0.009	0.043
Grade 3-4	8 (5.06%)	1 (2.08%)	0.688	0.964
Anemia				
Grade 1-2	64 (40.51%)	4 (8.33%)	<0.001	<0.001
Grade 3-4	0 (0.00%)	1 (2.08%)	0.233	1.000
Increased alanine transaminase				
Grade 1-2	18 (11.39%)	5 (10.42%)	1.000	0.997
Grade 3-4	2 (1.27%)	0 (0.00%)	1.000	1.000
Thyroid dysfunction				
Grade 1-2	19 (12.03%)	4 (8.33%)	0.606	0.361
Grade 3-4	0 (0.00%)	0 (0.00%)	None	None
Myocardial enzyme elevation				
Grade 1-2	0 (0.00%)	0 (0.00%)	None	None
Grade 3-4	0 (0.00%)	0 (0.00%)	None	None
Total				
Grade 1-2	85 (53.80%)	28 (58.33%)	0.622	0.835
Grade 3-4	10 (6.33%)	4 (8.33%)	0.743	0.451

*P value was adjusted by clinical factors, included age, gender, tumor location, differentiation, ECOG performance status, clinical T stage, clinical N stage, smoking history, alcohol consumption history, chemotherapy regimen, and cycles of neoadjuvant treatment.

### Surgery outcomes

Perioperative complications were summarized in **Table 3**. All patients achieved R_0_ resection in both groups. The nCT + ICIs group had a greater number of lymph node dissected (42.26 ± 18.47 vs. 31.19 ± 12.48, *P*=0.023), but this was accompanied by longer operation time (334.00 ± 170.2min vs. 279.60 ± 88.31min, *P*=0.020). However, there were no differences in the number of lymph node resection stations and the count of positive lymph node between the groups. Regarding surgical complications, the incidence rates in nCT + ICIs and nCRT + ICIs groups were as follows: pneumonia (5.70% vs 14.58%, *P*=0.317), respiratory failure (6.33% vs 6.25%, *P*=0.983), pneumothorax (2.53% vs 2.08%, *P*=0.997), anastomotic leakage (18.35% vs 12.50%, *P*=0.543), tracheal fistula (0.63% vs 2.08%, *P*=0.995), and chylothorax (3.16% vs 4.17%, *P*=0.911). In nCT + ICIs group, postoperative bleeding and acute kidney injury occurred in 1 and 2 patients, respectively. However, there were no statistically significant differences in the occurrence of various complications.

**Table 3:** Summary of surgery outcomes.

Surgery outcomes	nCT + ICIs (n=158)	nCRT + ICIs (n=48)	*P* value	Adjusted *P* value*
R0 resection rate	158 (100%)	158 (100%)	1.000	1.000
Lymph node resection number	42.26 ± 18.47	31.19 ± 12.48	<0.001	0.023
Lymph node resection station	10.23 ± 3.54	10.27 ± 1.69	0.070	0.079
Positive lymph node number	0.82 ± 2.06	0.52 ± 1.41	0.391	0.377
Operation time/min	334.00 ± 170.2	279.60 ± 88.31	0.020	0.020
Blood loss/ml	102.5 ± 28.13	78.13 ± 32.46	0.021	0.201
Perioperative Complication				
Pneumonia	9 (5.70%)	7 (14.58%)	0.062	0.317
Respiratory failure	10 (6.33%)	3 (6.25%)	1.000	0.983
Pneumothorax	4 (2.53%)	1 (2.08%)	1.000	0.997
Anastomotic leakage	29 (18.35%)	6 (12.50%)	0.508	0.543
Tracheal Fistula	1 (0.63%)	1 (2.08%)	0.413	0.995
Chylothorax	5 (3.16%)	2 (4.17%)	0.666	0.911
Postoperative Bleeding	1 (0.63%)	0 (0%)	1.000	1.000
Kidney injury	2 (1.27%)	0 (0%)	1.000	0.997
Perioperative death	0 (0%)	0 (0%)	1.000	1.000

*P value was adjusted by clinical factors, included age, gender, tumor location, differentiation, ECOG performance status, clinical T stage, clinical N stage, smoking history, alcohol consumption history, chemotherapy regimen, and cycles of neoadjuvant treatment.

### Comparisons of short and long-term outcomes

Tumor response outcomes was seen in **Table 4**. The ORR in nCT + ICIs and nCRT + ICIs groups was 73.4% and 79.2%, respectively. After adjusting for clinical factors, there was a significant statistical difference in the ORR between the two groups (OR: 2.888, 95%CI: 1.064-7.840, *P*=0.037). nCRT + ICIs group achieved a pCR rate of 52.1%, which was also higher than nCT + ICIs group’s 32.3% (OR: 3.946, 95%CI: 1.547-10.067, *P*=0.004).

**Table 4:** Summary tumor response.

Tumor response	nCT + ICIs (n=158)	nCRT + ICIs (n=48)	Before adjusted		After adjusted*	
			*P* value	OR (95%CI)	*P* value	OR (95%CI)
RECIST						
CR	10 (6.3%)	2 (4.2%)	0.736	0.644 (0.137-2.696)	0.930	0.908 (0.105-7.823)
PR	106 (67.1%)	36 (75.0%)	0.374	1.472 (0.729-3.026)	0.027	2.996 (1.136-7.902)
SD	39 (24.7%)	10 (20.8%)	0.700	0.803 (0.380-1.762)	0.109	0.440 (0.162-1.200)
PD	3 (1.9%)	0 (0%)	1.000	-	1.000	-
ORR	116 (73.4%)	38 (79.2%)	0.456	1.376 (0.633-2.893)	0.037	2.888 (1.064-7.840)
TRG						
Grade 0	51 (32.3%)	25 (52.1%)	0.017	2.280 (1.158-4.284)	0.004	3.946 (1.547-10.067)
Grade 1	35 (22.2%)	13 (33.3%)	0.559	1.305 (0.643-2.714)	0.482	1.429 (0.529-3.861)
Grade 2	34 (21.5%)	8 (16.37%)	0.544	0.729 (0.330-1.643)	0.595	0.742 (0.248-2.227)
Grade 3	38 (24.1%)	2 (4.2%)	0.002	0.137 (0.032-0.516)	0.001	0.051 (0.010-0.274)
**pCR rate**	51 (32.3%)	25 (52.1%)	0.017	2.280 (1.158-4.284)	0.004	3.946 (1.547-10.067)

*P value was adjusted by clinical factors, included age, gender, tumor location, differentiation, ECOG performance status, clinical T stage, clinical N stage, smoking history, alcohol consumption history, chemotherapy regimen, and cycles of neoadjuvant treatment.

**Figure 3:**
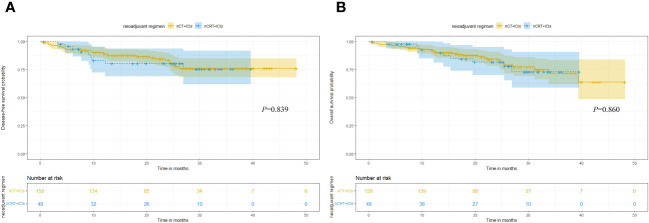
Kaplan-Meier survival analysis. **(A)** Comparison of DFS between nCT + ICIs group and nCRT + ICIs group. **(B)** Comparison of OS between nCT + ICIs group and nCRT + ICIs group.

**Figure 4:**
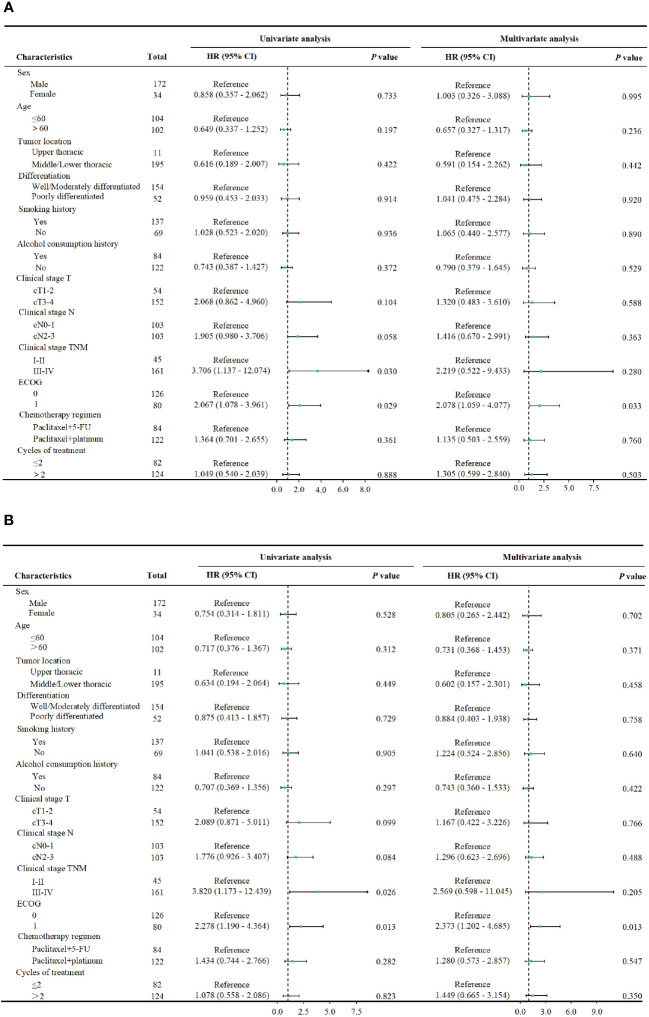
Cox proportional hazards model for univariate and multivariate analysis. **(A)** The association between pretreatment characteristics and DFS. **(B)** The association between pretreatment characteristics and OS.

In terms of long-term follow-up, the median follow-up time for nCT + ICIs group was 22.05 months (95%CI: 19.40-24.20), while it was 24.65 months (95%CI: 15.30-26.80) for the nCRT + ICIs group. The 2-year DFS were 83.21% (95%CI: 4.469-7.720) and 80.47% (95%CI: 9.304-15.914) in the two groups, respectively (*P*=0.839). The 2-year OS were 84.42% (95%CI: 5.253-7.560) and 81.70% (95%CI: 9.178-16.455) in the two groups, respectively (*P*=0.860). The survival curves for both groups were shown in **Figure 3**. Through univariate and multivariate Cox regression analysis, we found that patients with better ECOG performance status tended to have a more favorable DFS and OS compared to those with poorer ECOG performance status. While the chemotherapy regimen and the cycles of neoadjuvant therapy showed no significant impact on prognosis. The results of univariate and multivariate regression analyses were presented in **Figure 4**.

### The pattern of treatment failure

The sites of recurrence or metastasis for both groups were listed in **Table 5**. The recurrence patterns of both groups included regional, distant recurrence, or both. In the nCT + ICIs group, a total of 22 (13.92%) patients experienced recurrence, among whom 8 (5.06%) cases had regional recurrence, 11 (6.96%) cases had distant metastasis, and 3 (1.90%) cases had both regional recurrence and distant metastasis. In the nCRT + ICIs group, 9 (18.75%) patients experienced recurrence, with 1 (2.08%) case of regional recurrence, 6 (12.50%) cases of distant metastasis, and 2 (4.17%) cases of both regional recurrence and distant metastasis. There was no statistical difference between the two groups in terms of recurrence patterns (regional recurrence: *P*=0.251, distant metastasis: *P*=0.158, both: *P*=0.695).

**Table 5:** Summary of treatment failure patterns.

Failure patterns	nCT + ICIs (n=158)	nCRT + ICIs (n=48)	*P* value	*P* value*
**Regional/local**	8 (5.06%)	1 (2.08%)	0.376	0.251
Anastomosis recurrence	1 (0.63%)	0 (0.00%)	0.581	1.000
Mediastinal lymph node metastasis	7 (4.43%)	1 (2.08%)	0.138	0.270
**Distant**	11 (6.96%)	6 (12.50%)	0.222	0.158
Supraclavicar lymph node metastasis	2 (1.27%)	1 (2.08%)	0.679	0.996
Cervical lymph node metastasis	1 (0.63%)	1 (2.08%)	0.369	0.996
Peritoneal metastasis	1 (0.63%)	0 (0.00%)	0.581	1.000
Brain metastasis	2 (1.27%)	0 (0.00%)	0.434	0.999
Bone metastasis	0 (0.00%)	1 (2.08%)	0.069	1.000
Liver metastasis	2 (1.27%)	2 (4.17%)	0.202	0.433
Lung metastasis	3 (1.90%)	1 (2.08%)	0.935	0.548
**Both**	3 (1.90%)	2 (4.17%)	0.371	0.695

*P value was adjusted by clinical factors, included age, gender, tumor location, differentiation, ECOG performance status, clinical T stage, clinical N stage, smoking history, alcohol consumption history, chemotherapy regimen, and cycles of neoadjuvant treatment.

### Economic efficiency analysis

**Figure 5:**
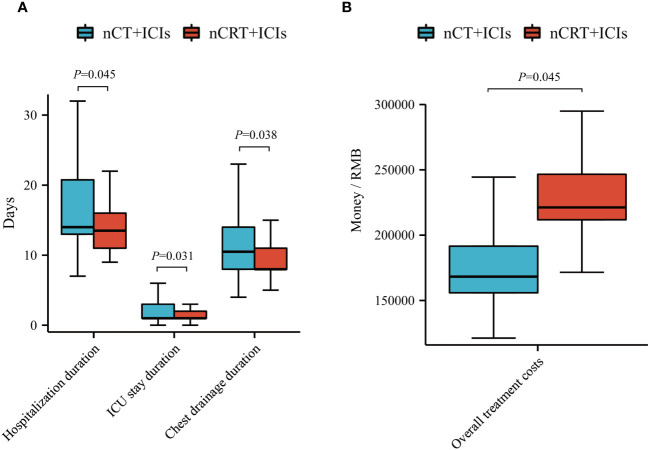
Economic efficiency analysis. **(A)** Compare hospitalization duration, ICU stay duration and chest drainage duration between nCT + ICIs group and nCRT + ICIs group. **(B)** Compare overall treatment costs between nCT + ICIs group and nCRT + ICIs group.

We compared the differences in hospitalization duration, ICU stay duration and chest drainage duration between the two groups. After adjusting for clinical factors, the nCT + ICIs group had longer hospitalization duration (19.20 ± 12.98 days vs 16.33 ± 10.61 days, *P*=0.045), ICU stay duration (2.37 ± 2.32 days vs 1.79 ± 1.95 days, *P*=0.031), and chest drainage duration (13.18 ± 9.55 days vs 10.96 ± 9.21 days, *P*=0.038) compared to the nCRT + ICIs group. However, there were no differences in the 30-day and 90-day readmission rates after discharge between the two groups. Additionally, the overall treatment costs in the nCRT + ICIs group were significantly higher than those in the nCT + ICIs group (188796.00 ± 107704.00 RMB vs 231808.00 ± 48067.00 RMB, *P*=0.045). Detailed information was provided in **Figure 5** and **Table 6**.

**Table 6:** Summary of readmission rate.

Readmission rate	nCT + ICIs (n=158)	nCRT + ICIs (n=48)	Before adjust		After adjust*	
			*P* value	OR (95%CI)	*P* value	OR (95%CI)
**30-day readmission**			0.392	2.040 (0.522-8.119)	0.581	1.864 (0.204-17.073)
Yes	5 (3.16%)	3 (6.25%)				
No	153 (96.84%)	45 (93.75%)				
**90-day readmission**			1.000	0.987 (0.281-3.623)	0.680	0.701 (0.130-3.787)
Yes	10 (6.33%)	3 (6.25%)				
No	148 (93.67%)	45 (93.75%)				

*P value was adjusted by clinical factors, included age, gender, tumor location, differentiation, ECOG performance status, clinical T stage, clinical N stage, smoking history, alcohol consumption history, chemotherapy regimen, and cycles of neoadjuvant treatment.

## Discussion

In this study, we retrospectively compared two neoadjuvant immunotherapy treatment models in terms of safety, efficacy, and economic costs. We found that both treatment models had manageable adverse events, and there were no differences in surgical complications. nCRT + ICIs achieved a higher ORR and pCR rate, but it did not lead to a significant improvement in 2-year OS and DFS when compared to nCT + ICIs. Moreover, the overall treatment costs were higher for nCRT + ICIs than nCT + ICIs group.

In the recent years, nCT + ICIs was one of the most extensively explored neoadjuvant regimen. According to recent prospective phase II clinical trials reported, the pCR rate can reach to 20.0%-40.0% (12–16), and toxicities are mostly grade 1-2, with satisfactory safety profiles. Furthermore, several retrospective studies have compared nCT + ICIs to traditional neoadjuvant chemotherapy or neoadjuvant chemoradiotherapy. Junfeng Liu et al. (17) found that the pCR rate in nCT + ICIs group was significantly higher than that in neoadjuvant chemotherapy group, with a similar occurrence of adverse events, and a longer 1-year DFS. Similarly, in other studies compared nCT + ICIs with neoadjuvant chemoradiotherapy (18–20), nCT + ICIs had a comparable safety profile and surgical complications to chemoradiotherapy. In addition, some researchers were exploring different neoadjuvant immunotherapy models, such as nCRT + ICIs. In these studies (8, 21), nCRT + ICIs achieved a higher pCR rate and demonstrated promising short-term efficacy. However, current researches are single-arm trials, and lack of studies directly comparing nCT + ICIs with nCRT + ICIs. Therefore, it remains unclear which neoadjuvant immunotherapy model is the optimal approach.

In our study, we found the common adverse events were hematologic toxicities in both nCT + ICIs and nCRT + ICIs groups, which was similar to previous reports (10, 13). However, there were slight variations in the types of adverse events. Anemia was most common in nCT + ICIs group, whereas nCRT + ICIs had a more significant impact on white blood cells and neutrophils. This difference might be attributed to a higher proportion of patients in nCT + ICIs group received prophylactic granulocyte colony-stimulating factors. This difference was only observed in grade 1-2 adverse events, with no disparities in grade 3 or severe toxicities.

As for the surgical aspect, several single-arm clinical trials have reported R0 resection rates after neoadjuvant nCT+ICIs ranging from 84.6% to 92.11% (22, 23), and after nCRT+ICIs ranging from 98% to 100% (15, 24). Similarly, in our study, both in the nCT+ICIs and nCRT+ICIs cohorts, the R0 resection rate reached 100%. Lymph node dissection is a crucial and difficult step in esophagectomy. The number of lymph node dissected is an indicator of surgical quality in esophagectomy. In the nCRT+ICIs group, the number of lymph nodes dissected was significantly less than that in the nCT+ICIs group, which may be related to the local tissue fibrosis induced by radiotherapy (25), increasing the difficulty of lymph node dissection. However, nCRT + ICIs group had advantages in surgical duration. This advantage might be associated with a higher percentage of MIE with robot-assisted in nCRT + ICIs group (27.08% vs 8.23%). As we all know, robot-assisted system offers more precise operation and clearer vision compared to thoracoscopy (26). Furthermore, the less number of lymph node dissected in nCRT + ICIs group is also related to the shorter surgical duration. Although the types and incidence of postoperative complications were similar in both groups, it’s worth noted that the rate of pneumonia in nCRT + ICIs group was nearly three times more than nCT + ICIs group. This could be related to radiation-induced lung injury (27). Overall, nCRT+ICIs increases the surgical difficulty to a certain extent, requiring higher levels of experience and technical proficiency from surgeons.

According to previous literature, the pCR after nCRT+ICIs ranged from 30.3%-55.6% (8, 24), and after nCT+ICIs ranged from 16.7%-34.21% (22, 28–30). The pCR rates reported from nCRT + ICIs studies are generally higher than those from nCT + ICIs studies. However, due to differences in the clinical and pathological characteristics of patients enrolled in diverse clinical trials, and long-term follow-up data are often lacking, the comparability is limited. In our study, the two cohorts were entirely balanced in terms of baseline characteristics, avoiding intergroup differences. The pCR rate in the nCRT+ICIs group was 52.1%, while in the nCT+ICIs group it was 32.3%. In this study, the immune checkpoint inhibitors we used were all PD-1 antibodies, with similar pharmacological effects. However, there were significant differences in chemotherapy regimens. Despite this, the pCR rates were similar to those reported previously, indicating that the impact of chemotherapy regimens on efficacy was minimal. We observed a significantly higher pCR rate in nCRT + ICIs group, which might be attributed to localized radiation can eliminate residual lesions that insensitive to immunochemotherapy. The achievement of pCR after neoadjuvant chemoradiotherapy is considered a prognostic factor (31). But in the context of neoadjuvant immunotherapy, the significance of pCR for long-term prognosis has not been demonstrated. Nevertheless, upon further comparison of long-term survival, we found that a higher pCR rate did not translate into advantages in survival. There was no difference in 2-year OS and DFS between the two groups. Moreover, both groups experienced treatment failure due to distant metastasis, but it remained lower than neoadjuvant chemoradiotherapy (25.3%) (4). There was no statistical difference between the two groups in this regard. Comparing the incidence of metastasis at different sites, nCRT + ICIs could further reduce the risk of mediastinal lymph node metastasis, but there was no statistical difference. Therefore, the necessity of adding additional radiotherapy to immunochemotherapy requires careful consideration, and awaits longer follow-up data.

In China, esophageal cancer predominantly occurs in economically disadvantaged regions (32), and the cost of treatment is a significant concern for many patients. In our study, we observed that nCT + ICIs resulted in longer hospital stays, longer ICU duration, and slower drainage tube removed, which might be associated with more lymph nodes dissected. As we all know, The more lymph nodes that were dissected, the longer the operational time required, and consequently, the larger the surgical field area. It led to increased postoperative thoracic drainage and delayed extubation time. Additionally, patients in the nCT+ICIs group had a higher incidence of anastomotic leakage postoperatively (18.35% vs. 12.50%), leading to prolonged healing time and delayed hospital discharge. However, from the time of diagnosis to hospital discharge, the overall costs for nCT + ICIs were substantially less than nCRT + ICIs. From economic perspective, nCT + ICIs might be more feasible and acceptable to the patient population.

To our knowledge, this is the first study to compare different neoadjuvant immunotherapy regimens. But our research still has certain limitations. Firstly, it is a retrospective study. Secondly, the sample is limited, and there is a lack of uniformity in treatment regimens, cycles, and surgical approaches, which may generate bias into the results. Lastly, the median follow-up duration is only 2 years, and as the follow-up period extends, it is needed to determine whether differences could appear in survival between the two groups.

## Conclusion

Overall, nCT + ICIs and nCRT + ICIs demonstrated similarities in terms of toxicities, perioperative complications, and patterns of recurrence. Despite the higher pCR rate associated with nCRT + ICIs, it did not lead to the improvement of 2-year OS and DFS. Moreover, it incurred higher treatment costs. Therefore, when formulating neoadjuvant immunotherapy strategies, comprehensive consideration is warranted.

## Data availability statement

The raw data supporting the conclusions of this article will be made available by the authors, without undue reservation.

## Ethics statement

The studies involving humans were approved by Sun Yat-sen university cancer center ethics committee. The studies were conducted in accordance with the local legislation and institutional requirements. Written informed consent for participation in this study was provided by the participants’ legal guardians/next of kin.

## Author contributions

XiZ: Conceptualization, Funding acquisition, Supervision, Visualization, Writing – original draft. GY: Data curation, Formal Analysis, Methodology, Software, Validation, Visualization, Writing – original draft, Writing – review & editing. HY: Data curation, Methodology, Project administration, Writing – review & editing. XuZ: Software, Visualization, Writing – original draft, Writing – review & editing. CZ: Data curation, Methodology, Writing – original draft. LT: Data curation, Writing – review & editing.

## Supplementary material

Supplementary Figure 1 The regimen of neoadjuvant treatment. **(A)** The regimen of PD-1 inhibitors in nCT + ICIs group and nCRT + ICIs group. **(B)** The regimen of chemotherapy drugs in nCT + ICIs group and nCRT + ICIs group.
