# Supplementary figures and images for: Comparison of neoadjuvant chemotherapy or chemoradiotherapy plus immunotherapy for locally resectable esophageal squamous cell carcinoma
Source: Front Immunol. 2024 May 8;15:1336798. doi: 10.3389/fimmu.2024.1336798 (PMC11109365; doi:10.3389/fimmu.2024.1336798)

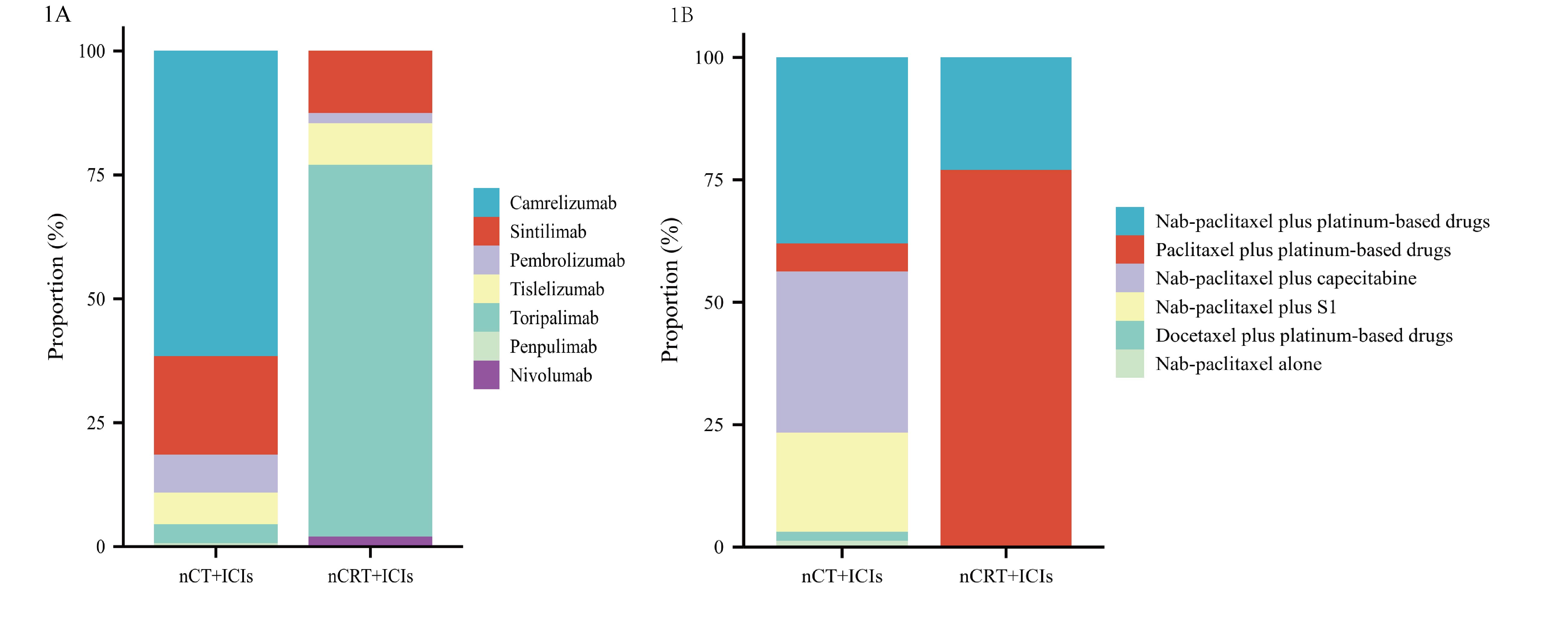

Supplement: Supplementary Figure 1 — The regimen of neoadjuvant treatment. (A) The regimen of PD-1 inhibitors in nCT + ICIs group and nCRT + ICIs group. (B) The regimen of chemotherapy drugs in nCT + ICIs group and nCRT + ICIs group. [file Image_1.jpeg]
