# Supplementary material for: Comparison of neoadjuvant chemotherapy or chemoradiotherapy plus immunotherapy for locally resectable esophageal squamous cell carcinoma
Source: Front Immunol. 2024 May 8;15:1336798. doi: 10.3389/fimmu.2024.1336798 (PMC11109365; doi:10.3389/fimmu.2024.1336798)
Supplement: Supplementary file 2 [file Table_1.docx]

**Supplementary Table 1. The detailed dose and usage of drugs in neoadjuvant regimens.**

| Type | Drugs | Dose | Usage |
| --- | --- | --- | --- |
| PD-1 inhibitors | Camrelizumab | At a dose of 200 mg | Every 3 weeks on day 1 |
|  | Pembrolizumab | At a dose of 200 mg | Every 3 weeks on day 1 |
|  | Sintilimab | At a dose of 200 mg | Every 3 weeks on day 1 |
|  | Tislelizumab | At a dose of 200 mg | Every 3 weeks on day 1 |
|  | Toripalimab | At a dose of 240 mg | Every 3 weeks on day 1 |
|  | Penpulimab | At a dose of 200 mg | Every 3 weeks on day 1 |
|  | Nivolumab | At a dose of 360 mg | Every 3 weeks on day 1 |
| Chemotherapy drugs | Paclitaxel | At a dose of 175 mg/m2 | Every 3 weeks on day 1 |
|  | Nab-paclitaxel | At a dose of 240 mg | Every 3 weeks on day 1 |
|  | Docetaxel | At a dose of 75 mg/m2 | Every 3 weeks on day 1 |
|  | Carboplatin | At an area under the curve of 5 | Every 3 weeks on day 1 |
|  | Cisplatin | At a dose of 50 mg/m2 | Every 3 weeks on day 1 |
|  | Capecitabine | At a dose of 1125 mg/m2 | Every 3 weeks on day 1 to 14 |
|  | S1 | At a dose of 40 mg/m2 | Every 3 weeks on day 1 to 14 |
| Radiotherapy | - | 40Gy/20 fractions | 5 days per week |
